# Nanoarchitectonics for High Adsorption Capacity Carboxymethyl Cellulose Nanofibrils-Based Adsorbents for Efficient Cu^2+^ Removal

**DOI:** 10.3390/nano12010160

**Published:** 2022-01-03

**Authors:** Rongrong Si, Yehong Chen, Daiqi Wang, Dongmei Yu, Qijun Ding, Ronggang Li, Chaojun Wu

**Affiliations:** State Key Laboratory of Biobased Material and Green Papermaking, Qilu University of Technology (Shandong Academy of Sciences), Jinan 250353, China; srr18238657108@163.com (R.S.); yudongmei197@163.com (D.Y.); xianshengding@qlu.edu.cn (Q.D.); lrg923@163.com (R.L.)

**Keywords:** carboxymethylation, cellulose nanofibrils, aerogel, adsorption

## Abstract

In the present study, carboxymethyl cellulose nanofibrils (CMCNFs) with different carboxyl content (0.99–2.01 mmol/g) were prepared via controlling the ratio of monochloroacetic acid (MCA) and sodium hydroxide to Eucalyptus bleached pulp (EBP). CMCFs-PEI aerogels were obtained using the crosslinking reaction of polyethyleneimine (PEI) and CMCNFs with the aid of glutaraldehyde (GA). The effects of pH, contact time, temperature, and initial Cu^2+^ concentration on the Cu^2+^ removal performance of CMCNFs-PEI aerogels was highlighted. Experimental data showed that the maximum adsorption capacity of CMCNF30-PEI for Cu^2+^ was 380.03 ± 23 mg/g, and the adsorption results were consistent with Langmuir isotherm (R^2^ > 0.99). The theoretical maximum adsorption capacity was 616.48 mg/g. After being treated with 0.05 M EDTA solution, the aerogel retained an 85% removal performance after three adsorption–desorption cycles. X-ray photoelectron spectroscopy (XPS) results demonstrated that complexation was the main Cu^2+^ adsorption mechanism. The excellent Cu^2+^ adsorption capacity of CMCNFs-PEI aerogels provided another avenue for the utilization of cellulose nanofibrils in the wastewater treatment field.

## 1. Introduction

With the rapid development of the social economy, heavy metal pollution has become a thorny and crucial problem in modern society [1]. Anthropogenic activities such as mining, smelting, oil refining, and the manufacture of paint, release large amounts of these toxic and dangerous chemicals into the environment [2]. Of all kinds, Cu^2+^ is considered to be a major contaminant in water and can accumulate in the human liver, causing severe hemolysis and anemia. Therefore, the removal of Cu^2+^ from wastewater in an effective manner has become an important current issue [3].

There are many ways to solve the problem of heavy metal ion pollution in wastewater, mainly including chemical precipitation, ion exchange, ultrafiltration, flocculation, electrodialysis, adsorption, and reverse osmosis, etc. [4]. Among these methods, adsorption is very popular due to its high removal efficiency, flexibility in design, and low cost [5]. The adsorbents usually include activated carbon, clay, biochar, and polymers, etc. [6]. Although these adsorbents have high adsorption capacities for some heavy metal ions, they still present some disadvantages, such as unsatisfactory non-biodegradability, high cost of preparation or renewable energy, and secondary pollution. Therefore, it is necessary to find a kind of adsorbent with high adsorption capacity to solve this problem.

Recently, cellulose, as the most common structural amphiphilic renewable polymer resource in the biosphere, has been proven to have good adsorbent adsorption performance [7,8]. Many carboxylated nanocelluloses have been used in the removal of heavy metal ions, such as TO-CNF obtained by TEMPO oxidation [9], CNC obtained by Fe^2+^/H_2_O_2_ oxidation [10], and carboxymethylated nanocellulose [11]. Among them, carboxymethyl cellulose (CMCs), a cellulose derivative with a high carboxyl group content obtained by the alkalization and etherification of cellulose [8], is one of the most promising substrates for aerogels on account of its economic benefits and non-toxic natural polymers [11]. However, the abundant carboxymethyl groups make them very hydrophilic and thus limit their practical applications in aqueous environments. The separation of CMC-based adsorbents from water has become a difficult problem, and affects the regeneration and recycling of the adsorbent [12]. Therefore, it is an imperative problem to preparena CMC-based adsorbent which is stable in water and has cyclic performance. Assembling individual fibers into aerogel/hydrogel is a better way to easily separate from bulk solutions as aerogel consists of interconnected porous solid materials. Li et al. [11] used Al^3+^ as a crosslinking agent to obtain CMC-Al beads for removing heavy metal ions. As well, there are a few reports of combining CMC with other biological macromolecules for removing heavy metal ions, such as CMC-chitosan [13] and CMC-sodium alginate [14]. Li et al. [15] obtained the NFC solution through TEMPO oxidation, and then achieved physically crosslinked network NFC/PEI composite hydrogel (NPH) through electrostatic combination with PEI (polyethyleneimine) solutions, which showed good adsorption effect for Cu^2+^ and Pb^2+^. However, rare studies have reported on grafted PEI with CMCNFs.

Branched PEI has plenty of amino groups, and usually were selected to fabricate adsorbents [16]. In this article, CMCFs-PEI aerogels were obtained using the crosslinking reaction of polyethyleneimine (PEI) and CMCNFs with different carboxyl content. Furthermore, their Cu^2+^ removal performance was systematically investigated under different conditions. After that, the removal mechanism of Cu^2+^ removal was demonstrated using FTIR (Fourier transform infrared spectroscopy) and XPS analysis. The adsorption and desorption characteristics of the aerogel were also detected.

## 2. Experimental

### 2.1. Methods and Materials

Materials: Eucalyptus bleached pulp (EBP, Jinan, Shandong Province, China), sodium hydroxide (NaOH, AR grade, Jinan, Shandong Province, China), monochloroacetic acid (MCA, Jinan, Shandong Province, China), anhydrous methanol(Jinan, Shandong Province, China), glutaraldehyde (GA, 50% in water, Aladdin), PEI (polyethylenrimine, 70000 Mw, Macklin), copper nitrate (Cu (NO_3_)_2_ (1000 mg/L), Beijing, China), hydrochloric acid (HCl, 37%, Jinan, Shandong Province, China), ethylenediaminetetraacetic acid (EDTA, Jinan, Shandong Province, China).

### 2.2. Preparation of Carboxymethyl Cellulose Nanofibers (CMCNFs) Dispersions

During the reaction, the quality ratio of eucalyptus bleached pulp (EBP) to NaOH was 1:1 (*w*/*w*). In order to obtain CMCNFs dispersions with different carboxylate contents, reagents with different MCA (10, 20, 30 g) were selected.

First, dry weight EBP (20 g) was blended with 100 mL NaOH solution (5 mol/L) in a sealed bag and the mixture was vigorously rubbed at room temperature for one hour. The MCA solution (40% MCA/deionized water *w*/*v*) was put in a sealed bag slowly and heated it to 65 °C for 2 h. The treated wood fibers are then repeatedly rinsed with the ethanol mixture until the pH of the filtrate was neutral. After that, the carboxymethylated EBP was treated with a high pressure homogenizer. The samples were denoted as CMCNF10, 20, 30 (10, 20, 30 were the amount of MCA).

### 2.3. Preparation of CMCNFs-PEI Aerogel

A total of 50 mL CMCNFs suspension (0.5 wt%) reacted with 50 mL GA (0.5%) solution at 50 °C for 0.5 h to crosslinked CMCNFs and GA. Subsequently, 50 mL of PEI solution (2% in water) was added to the mixture. After mixing and stirring for 10 min, the product was centrifuged and washed thoroughly with deionized water 3 times, and then freeze-dried to obtain CMCNFs-PEI aerogel.

### 2.4. Characterization

Both wood fibers, CMCNFs (freeze-dried aerogel) and CMCNFs-PEI aerogels, were characterized by FTIR (Prestige21, Shimadzu Corporation, Karlsruhe, Germany). The content of carboxylate in CMCNFs was determined by standard conductance titration (DDSJ-319L, Shanghai, China). The morphology of CMCNFs was characterized by atomic force microscope (AFM, Brooker, Karlsruhe, Germany). Scanning electron microscope (SEM, Regulus 8220, Tokyo, Japan) was used to observe the form of CMCNFs-PEI aerogel. The crystalline structure of EBP and CMCNFs was measured by X-ray diffraction (XRD, Bruker, Karlsruhe, Germany). The carboxyl content of the prepared CMCNFs was determined by conductivity meter (DDSJ-318, Shanghai, China). Thermogravimetric analysis (TGA) of CMCNFs was performed using a synchronous thermal analyzer (STA449, Selb, Germany) under inert (N_2_) atmosphere heated continuously from room temperature (RT) at 10 °C/min to 600 °C. Besides, X-ray photoelectron spectroscopy (XPS, ESCALABXi+, New York, NY, USA) was analyzed for the surface chemical compositions of CMCNF30-PEI aerogel and CMCNF30-PEI loaded Cu^2+^.

### 2.5. Adsorption Experiments for Cu^2+^ Adsorption by Three Aerogels

The adsorption kinetics and isotherms of Cu^2+^ by CMCFs-PEI were investigated by batch adsorption experiments. Add aerogels (0.05 g) and Cu^2+^ solution (50 mL) to a 100 mL flask. Then put the flask in Temersionoxcillator registrarion (NoKi) at 25 °C to reach the adsorption equilibrium. When the pH exceeded 6.5, Cu^2+^ ions could be converted to Cu (OH)_2_ precipitation [14]. The effect of initial pH values in the range of 2–6 was investigated, in which the pH of the Cu^2+^ solution was controlled by 1 M NaOH/1 M HCl solution. For the kinetics study, the effect of contact time from 0 to 8 h was investigated at optimal pH. The adsorption isotherm experiments were carried out in the concentration range of 20~400 mg/L metal ions. The residual concentration of Cu^2+^ were determined by flame atomization atomic absorption spectrometry (AAS, GGX-600, China), the equilibrium adsorption capacity (*q_e_*, mg/g) and removal rate were calculated according to (Equation (1)) [17].

(1) $$q_{e}=\frac{\left(C_{0}-C_{e}\right)\times V}{m}$$

(2) $$R=\frac{\left(C_{0}-C_{e}\right)}{C_{0}}\times 100\%$$

where *C*_0_ is the initial concentration of Cu^2+^ (mg/L) and *C_e_* is the concentration of Cu^2+^ at equilibrium time; *V* (mL) is the volume of the solution and *m* (mg) is the aerogel dose.

### 2.6. Adsorption Kinetics and Isotherms

Pseudo-first-order and second-order kinetic models were used to simulate the adsorption kinetic data of Cu^2+^ on CMCNFs-PEI, and the expression is found in Equations (3) and (4) [18]:

(3) $$\log\left(q_{e}-q_{t}\right)=logq_{e}-\frac{k_{1}t}{2.303}\;\mathrm{or}\;\ln\left(q_{e}-q_{t}\right)=lnq_{e}-k_{1}t$$

(4) $$\frac{t}{q_{t}}=\frac{1}{k_{2}q_{e}{}^{2}}+\left(\frac{1}{q_{e}}\right)t$$

where *q_t_* is the adsorption capacity after time *t* and *q_e_* is the saturated adsorption capacity of Cu^2+^; *k*_1_ (min^−1^) and *k*_2_ (g/(mg min)) are rate constants of pseudo-first- and second-order kinetics, respectively.

The adsorption isotherms of Cu^2+^ adsorption by CMCNFs-PEI aerogel; the Langmuir and Freundlich adsorption isotherm models were used to analyze the experimental data, which are represented as Equations (5) and (6) [19]:

(5) $$\frac{C_{e}}{Q_{e}}=\frac{C_{e}}{Q_{m}}+\frac{1}{Q_{m}b}$$

(6) $$lgQ_{e}=lgK_{f}+\frac{1}{n}lgC_{e}$$

where *Q_e_* (mg/g) is the equilibrium adsorption capacity, *C_e_* (mg/L) is the Cu^2+^ solution concentration at equilibrium, *Q_m_* (mg/g) is the maximum adsorption capacity and *b* is the Langmuir adsorption constant related to adsorption energy, *K_f_* and *n* are the Freundlich adsorption constants which indicate the capacity and intensity of the adsorption, respectively.

Thermodynamic parameters such as free energy, enthalpy change and entropy change are determined by thermodynamic equations, as follows (7) and (8) [20]:

(7) $$Ln\left(K_{d}\right)=\frac{-\Delta H}{\mathrm{RT}}+\frac{\Delta S}{R}$$

(8) ΔG=ΔH − TΔS

where *K_d_* (mL/g) is the equilibrium constant, *R* is the gas constant (8.314 J/mol/K), and *T* (*K*) is the absolute temperature.

### 2.7. Cycle Testing of CMCNF_S_-PEI

In order to evaluate the cycling stability of the aerogel, the adsorption-desorption cyclic experiments were carried out. Cu^2+^ was adsorbed by CMCNF30-PEI aerogel, then immersed in 0.05 M EDTA solution and stirred at 25 °C for 3 h to remove Cu^2+^. The aerogel was then immersed in distilled water several times to elute all the salt until a pH of about 7 was reached before another adsorption process was carried out.

## 3. Results and Discussion

### 3.1. Characterization of the Aerogel

**Figure 1:**
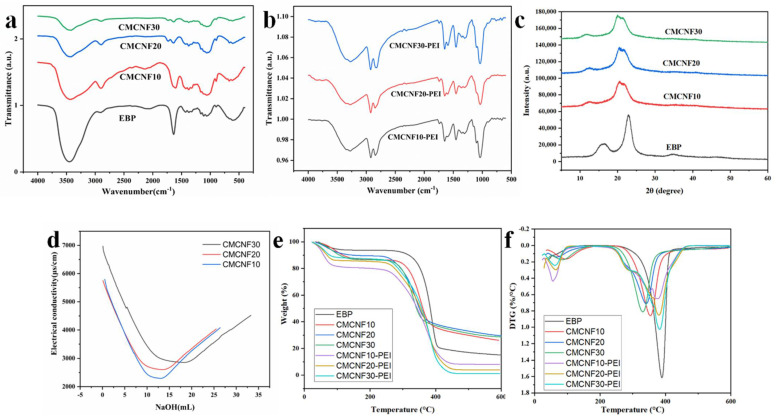
FTIR spectra of (**a**) EBP, CMCNFs, (**b**) CMCNFs-PEI, (**c**) XRD spectra of EBP and CMCNFs, (**d**) illustration of approach used for determination of carboxyl group in CMCNFs, (**e**) TGA, and (**f**) DTG curves of all samples.

The FTIR spectra of EBP, CMCNFs, and CMCNFs-PEI are presented in Figure 1a. A comparison of the FTIR results reveals that new peaks appeared in CMCNFs after carboxymethylation. For EBP, the peak at 3400 cm^−1^ corresponds to the stretching vibration of O–H groups, and the peak at 1370 cm^−1^ and 1322 cm^−1^ belong to C-H vibration [21], whereas in the FTIR spectra of the CMCNF10 sample, the stretching vibration of the carbonyl (C=O) present in the carboxylate groups (COO^−^) was observed at 1606 cm^−1^. However, CMCNF20 and CMCNF30 have a stretching vibration at 1740 cm^−1^ (−COOH), which may be due to higher carboxyl content. To prove the successful grafting of PEI onto CMCNFs, the FTIR spectra of CMCNFs-PEI aerogels are shown in Figure 1b. Compared to CMCNFs, there are many new adsorption peaks appearing at 1453 cm^−1^, 1592 cm^−1^, and 1648 cm^−1^ associated with the amino groups on CMCNFs-PEI aerogels. Also, the occurrence of -CH_2_- stretching vibrations at 2923 cm^−1^ and 2848 cm^−1^ also can prove it. The adsorption peak at 1740 cm^−1^ should be contributed from stretching C=O groups, which appeared in three CMCNFs-PEI aerogels.

The XRD was used to analyze the effect of carboxymethylation on the crystal structure of CMCNFs (Figure 1c). Three peaks were observed at 2θ = 15.72°, 22.5°, and 34.5° in EBP. This is representative of EBP which is typical of cellulose I. For CMCNFs, the 2θ = 22.5° diffraction peak disappeared, and the 2θ = 20.5° diffraction peak corresponding to the alkali cellulose became obvious. At 2θ = 15.72°, the diffraction peak became weak and disappeared at 2θ = 34.5°. After treatment with 5 mol/L NaOH solution, the crystal lattice changed greatly, which is called intracellular swelling. There are two kinds of intracellular swelling. One is where the solvent enters the intracellular cell and increases the distance between macromolecular chains of cellulose, which is also called separate swelling. The other involves the formation of a compound with cellulose, which then changes the cellular structure of cellulose, and the cellular structure of primary cellulose is transformed into the crystalline structure of alkali cellulose [22].

The thermal degradation onset temperature (T_0_) and maximum decomposition temperature (T_max_) are shown in Table 1. During the process from RT to 100 °C, due to the loss of water, the four substances have a slight quality degradation. The T_0_ and T_max_ of the EBP were about 276.5 and 388.78 °C, respectively; thus, the CMCNFs exhibited a lower T_0_ and T_max_. The NaOH/ chloroacetic acid system introduced a large amount of carboxyl groups, and at neutral pH, the hydrogen bonds were disrupted and the molecular chains became less constrained. A higher carboxyl content lowered the T_0_ and T_max_, for example, T_0_ and T_max_ of CMCNF30 (232.96 and 330.78 °C) was lower than that of CMCNF10 (267.99 and 353.76 °C). The carboxyl group on the surface of the CNCNFs increased, and the thermal stability gradually decreased because of the more thermally unstable carboxyl groups insertion of the CNF surfaces. The more the carboxyl groups on the nanofibrils, the lower the T_0_ and T_max_.

**Figure 2:**
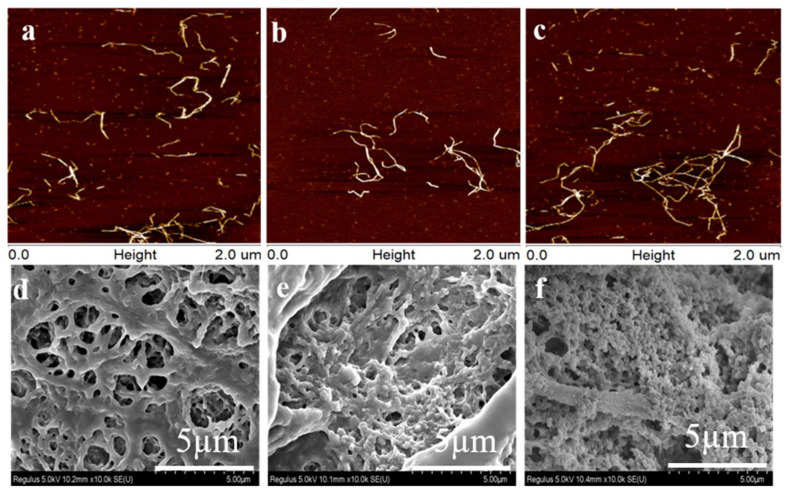
AFM images for dispersions of (**a**) CMCNF10, (**b**) CMCNF20, (**c**) CMCNF30; SEM images for aerogel of (**d**) CMCNF10-PEI, (**e**) CMCNF20-PEI, (**f**) CMCNF30-PEI.

The surface morphology of CMCNFs was examined by AFM (Figure 2a–c) and CMCNFs-PEI was examined by SEM (Figure 2d–f). As a result, it can be clearly seen that there is a larger aspect ratio. From the SEM of CMCNF_S_-PEI aerogel, it can be seen that there are pore structures in the typical 3D network. This can be used as a transport channel for heavy metal ions from the aqueous medium to enter the interior of the aerogel network through the rich pore structure. At the same time, it can also improve contact chance of Cu^2+^ with CMCNFs-PEI aerogel. When the adsorbent contacts the wastewater, water molecules can penetrate into the interior as soon as possible to achieve the effect of rapid adsorption. It may be that the water solubility of CMCNF30 increased with the increase of carboxyl group content, leading to the different surface morphology of CMCNF30-PEI. Compared with other heavy metal adsorbents, this aerogel has attracted extensive attention in the field of water treatment due to its unique 3D structure.

**Figure 3:**
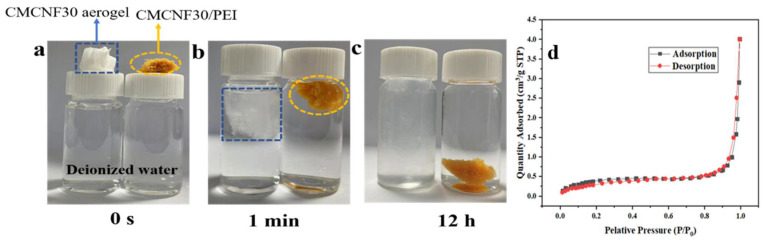
CMCNF30 and CMCNF30/PEI aerogel in deionized water for (**a**) 0 s, (**b**) 1 min, (**c**) 12 h; (**d**) adsorption–desorption isotherm of CMCNF30-PEI aerogel at 77K.

A total of 0.03 g of CMCNF30 aerogel obtained by freeze drying of CMCNF30 suspension was weighed and placed into 20 mL of deionized water for a hydrophilicity test. As shown in Figure 3, from the appearance, the color of CMCNF30 aerogel is white. After PEI modification, the color of CMCNF30/PEI aerogel turned yellow due to Schiff base reaction. CMCNF30/PEI aerogel of the same quality was used for comparison. The volume of both CMCNF30 and CMCNF30/PEI aerogels increased due to water absorption one minute after the aerogels were placed in deionized water. After 12 h, CMCNF30 aerogel dissolved in water, however, CMCNF30/PEI aerogel did not changed. This proves that the hydrophilicity of CMCNF decreases after PEI modification.

Figure 3d shows the N_2_ adsorption–desorption curve of the sample at a temperature of 77 K. The curve of CMCNF30-PEI aerogel shown in the figure is type four, which also verifies that the nanoparticles have a mesoporous structure. As can be seen from Table 2, the specific surface area and average pore size of CMCNF10-PEI increased from 0.82 to 1.52 m^2^/g, and the average pore size increased from 92.85 to 163.3 Å. The adsorption of P/P_0_ between 0–0.6 representing micropore (<20 Å) is negligible. The adsorption then increased rapidly, when P/P_0_ was between 0.6–1.0, representing mesoporous structures (20–500 Å) and indicating the presence of mesopores in CMCNF30-PEI, which provided a positive effect for the adsorption of heavy metals.

### 3.2. Cu^2+^ Adsorption

#### 3.2.1. Adsorption Kinetics of CMCNFs-PEI Aerogel

**Figure 4:**
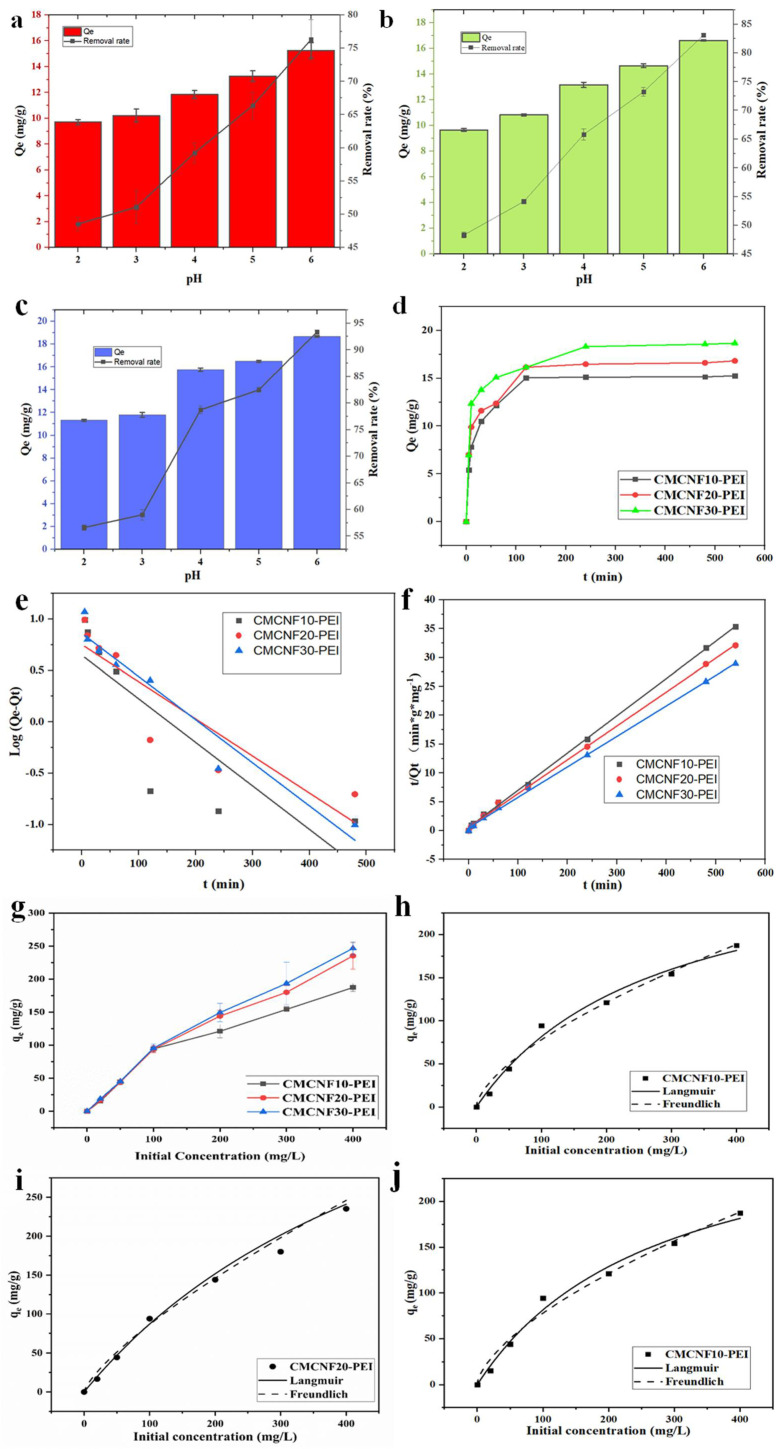
Effect of initial pH on Cu^2+^ adsorption; (**a**) CMCNF10-PEI (**b**) CMCNF20-PEI (**c**) CMCNF30-PEI. (**d**) Contact time removal capacity for Cu^2+^ adsorption (pH = 6, *C*_0_ = 20 mg/L, *T* = 25 °C). (**e**) Plots of pseudo-first-order, and (**f**) pseudo-second-order of CMCNF10-PEI, CMCNF20-PEI, CMCNF30-PEI. (**g**) Effect of different initial concentration on Cu^2+^ adsorption of three CMCNFs-PEI (pH = 6, *t* = 24 h, *T* = 25 °C). (**h**) CMCNF10, (**i**) CMCNF20-PEI, (**j**) CMCNF30-PEI isotherm model for Cu^2+^ adsorption.

We attempted to understand the Cu^2+^ removal performance of CMCNFs-PEI with pH and time. As presented in Figure 4a–c, due to the presence of amino groups on CMCNFs-PEI, the surface charge changes with the change of pH, thereby affecting the adsorption capacity. With the increase of pH, adsorption capacity also increases. Owing to the protonation of amino groups, the absorbent showed low adsorption capacity to Cu^2+^ at low pH conditions. When at a pH of six, the adsorption capacity increased to the maximum. It is noticeable that the adsorption capacity of aerogels increases as the carboxyl content of aerogels increases. According to the experimental data, the content of CMCNF10-PEI increased from 15.26 to 18.67 mg/g for CMCNF30-PEI. Therefore, the adsorption performance of CMCNFs-PEI for Cu^2+^ was studied under the condition of a pH of six.

To understand the basic kinetics of Cu^2+^ removal by CMCNFs-PEI, we evaluated the relationship between contact time (t) and removal capacity (Q_t_) between exposure time 0 to 540 min (Figure 4d,e). The removal rate increased rapidly at the beginning of the experiment and then reached equilibrium after 120 min. Two simplified kinetic models can be used to fit the experimental data of the adsorption process. The fitting results of adsorption kinetics for Cu^2+^ ion adsorption by CMCNFs-PEI aerogels is exhibited in Table 2. The non-linear correlation coefficient (R^2^) values for the second-order are higher than first-order, which indicates that the Cu^2+^ removal by CMCNFs-PEI is a rate-limiting and chemisorption process.

#### 3.2.2. Adsorption Isotherms of CMCNFs-PEI Aerogel

In order to confirm the interaction between CMCNFs-PEI and Cu^2+^, and that the maximum adsorption capacity of CMCNFs-PEI is the key to evaluate its removal performance, we studied the relationship between the initial Cu^2+^ concentration and equilibrium removal capacity of CMCNFs-PEI.

**Figure 5:**
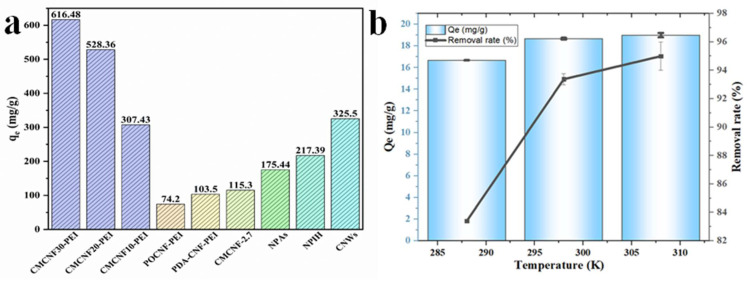
(**a**) The maximum adsorption capacity for Cu^2+^ with different cellulose-based adsorbents was compared with CMCNFs-PEI aerogels: Cu^2+^ adsorption capacity in this work, pomelo peel carboxylated cellulose nanofibers-PEI (POCNF-PEI) [24]; polydopamine-CNF-PEI (PDA-CNF-PEI) [25]; carboxymethylated CNFs (CMCNF-2.7) [26]; NFC/PEI composite aerogel (NPAs) [15]; NFCs/poly(2-(dimethylamino) ethyl methacrylate) interpenetrating network hydrogels (NPIHs) [27]; cellulose nanowhiskers (CNWs) [28]. (**b**) Effect of temperature on the Cu^2+^ adsorption of CMCNF30-PEI.

The removal results of the CMCNFs-PEI with different initial concentrations of Cu^2+^ are shown in Figure 4g–i. The adsorption isotherm parameters are displayed in Table 3. For CMCNF10-PEI, *R*^2^ > 0.98; for other adsorbents (CMCNF20-PEI, CMCNF30-PEI), *R*^2^ > 0.99. The results show that both Langmuir and Freundlich models can fit the adsorption isotherm well. The Langmuir model showed that the active adsorption sites were uniformly distributed on the surface of the aerogel, and the adsorption of Cu^2+^ ions occurred at the binding sites of functional groups and the surface, forming a monolayer [22]. However, Freundlich isotherm is used in the low to intermediate adsorbate concentration range [23]. The maximum Langmuir adsorption capacity for Cu^2+^ on CMCNF10-PEI aerogel was determined to be 307.43 mg/g. The adsorption capacity increased with the increase of carboxyl group content. The maximum adsorption capacity of CMCNF30-PEI was 616.48 mg/g, which was considered to be a super high capacity. This may be due to the carboxyl group and the amino group acting as electrostatic attraction and chelation to Cu^2+^, respectively. In Figure 5b, we provide a comparison between the current CMCNFs-PEI aerogels and other related Cu^2+^ adsorbents presented in the literatures. Compared with previous reported results, the adsorption ability of CMCNFs-PEI aerogels for Cu^2+^ are much higher.

#### 3.2.3. Thermodynamics of CMCNF30-PEI Adsorption

Thermodynamic parameters associated with the adsorption of Cu^2+^ using CMCNF30-PEI are presented in Figure 5b and Table 4. In this section, the adsorption capacity and removal efficiency of CMCNF30-PEI at a pH of six and the initial Cu^2+^ concentration of 20 mg/L at different temperatures (288, 298 and 308 K) were investigated. It can be seen from the figure that with the increase of temperature, both adsorption capacity and removal efficiency increase. ∆G > 0 and decreases with the increase of temperature, indicating the increasing spontaneity of the adsorption. Therefore, the adsorption of Cu^2+^ using the aerogel appears to be an irreversible and spontaneous endothermic process.

#### 3.2.4. Adsorption Mechanism of CMCNFs-PEI on Cu^2+^

**Figure 6:**
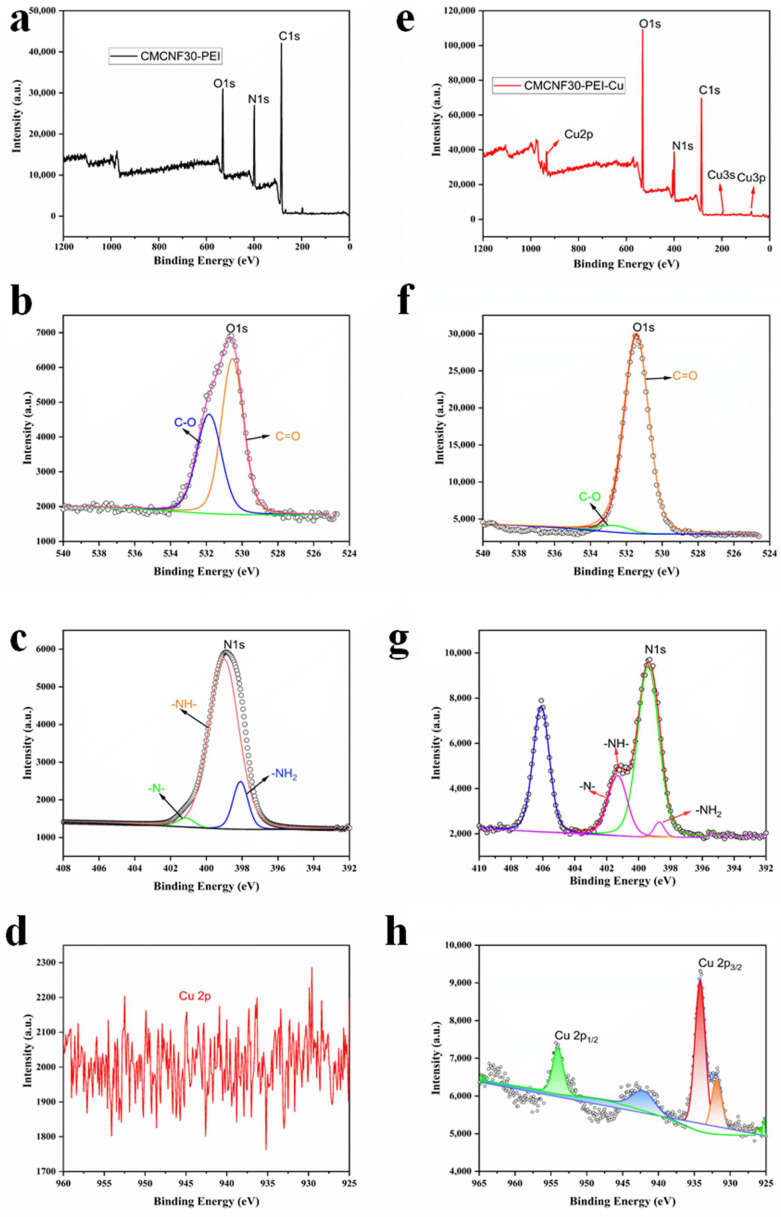
(**a**) Wide-scan XPS spectrum and (**b**–**d**) high-resolution core-level spectrum of CMCNF30-PEI, (**e**) wide-scan XPS spectrum and (**f**–**h**) high-resolution core-level spectrum of CMCNF30-PEI after Cu^2+^ adsorption.

XPS was used to study the elemental composition of CMCNF30-PEI and CMCNF30-PEI loaded Cu^2+^ ions (Figure 6a–h). Obviously, several new peaks appeared, assigned to Cu 2p doublet (Cu 2p_1/2_ and Cu 2p_3/2_), Cu 3s, and Cu 3p, which confirms the presence of Cu^2+^ on CMCNF30-PEI. In addition, the detailed XPS spectra of different elements at high resolution were deconvolved to evaluate the contribution of each component. As shown in Figure 6b, before adsorption, the N 1s peak could be fitted into the three components of 398.15, 399, and 400.15 eV corresponding to -N-, -NH- and -NH_2_, respectively. The high resolution XPS O 1s core-level spectrum of CMCNF30-PEI (Figure 6c) can be divided into two components at 530.5 eV and 531.8 eV, which were assigned to C-O and C=O, respectively. After Cu^2+^ ions were adsorbed (Figure 6f), the peaks of N 1s shifted to 398.7 eV, 399.3 eV, and 401.3 eV, and proved that all three amino groups are involved in the binding of Cu^2+^ ions [29]. At the same time, a new peak of 406.1 eV appeared in the N 1s spectra, which was due to the nitrogen in the amino groups sharing a lone electron pair to form the metal complex. In Figure 6g, for the O 1s spectra of CMCNF30-PEI aerogel, the C-O and C=O components increased to 532.9 eV and 531.5 eV, which is due to the electrons in the oxygen atom giving the oxygen of the carboxylic group a shared bond with the Cu^2+^ ions. In the expanded spectrum shown in Figure 6h, two characteristic peaks for the energy levels of Cu 2p_1/2_ (934.25 eV) and Cu 2p_3/2_ (954.15 eV) are visible [30]. In general, oxygen atoms and nitrogen atoms in CMCNF30-PEI have significant contributions to Cu^2+^ ion adsorption. The XPS spectra provided direct evidence of Cu^2+^ adsorption on CMCNF30-PEI via chemisorption.

**Figure 7:**
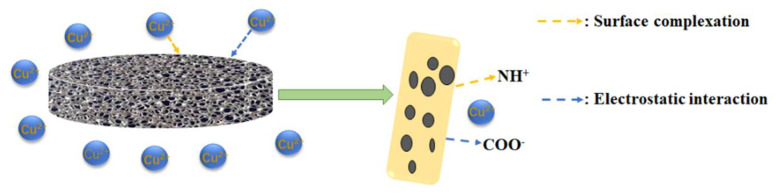
Schematic illustration of the adsorption mechanism of the CMCNFs-PEI.

The adsorption mechanism of the CMCNFs-PEI for Cu^2+^ derived from the data presented in the present study is shown in Figure 7. XPS results show that both N and O atoms have a certain effect on Cu^2+^. Among them, -COOH plays the role of electrostatic attraction, and functional groups such as -NH and -NH_2_ serve as a ligand for producing ligand–metal surface complexes, thus achieving the effect of Cu^2+^ removal.

#### 3.2.5. Desorption and Reusability

**Figure 8:**
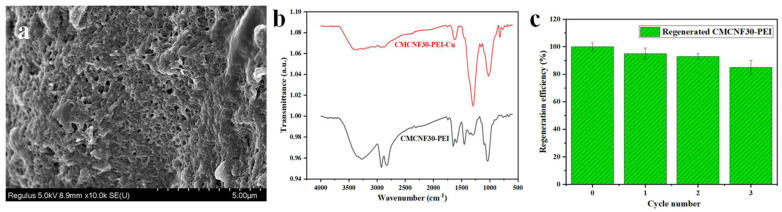
(**a**) SEM image of CMCNF30-PEI after adsorbed Cu^2+^. (**b**) FTIR spectra of CMCNF30-PEI aerogel and CMCNF30-PEI aerogel loaded with Cu^2+^. (**c**) Three adsorption cycles of CMCNF30-PEI aerogel for Cu^2+^ solution.

The ideal aerogel should have high adsorption capacity and have good regeneration and recycling properties, which are an important index of the practical application of water remediation. After adsorbtion with Cu^2+^ (Figure 8a), as can be clearly seen, the internal structure remained 3D reticulated, but the channels became tense. From FTIR spectra (Figure 8b), CMCNF30-PEI aerogel showed that the positions of characteristic peaks related to amino and carboxyl groups changed after Cu^2+^ adsorption. In order to explore the recycling ability of CMCNF30-PEI, 0.05 M EDTA solution was used as the desorption agent. Figure 8c shows the performance of CMCNF30-PEI for Cu^2+^ adsorption during three cycles. Although the regeneration efficiency decreased slightly with the cycle time, it still retained 85% after three adsorption–desorption cycles, indicating the excellent recyclability and reusability of the developed aerogel. The stability test showed the excellent cycling ability of the current test, thus it can be applied to practical wastewater treatment.

#### 3.2.6. Comparison with Reported Studies

The final adsorption properties of CMCNF30-PEI were evaluated and presented in Table 5 for comparison with reported data. The maximum adsorption capacity of CMCNF30-PEI for Cu^2+^ can reach 588.26 mg/g; 3-dialdehyde nano-fibrillated cellulose (DNFCs), TO-CNF/PVA/PEI nanoparticles, Fe_3_O_4_@zeolite NaA and composite from cellulose nanocrystals of Almond Prunus dulcis shell (CPCNCs) are much higher. Having excellent cycling performance as well, CMCNF30-PEI may play an important role in practical water treatment.

## 4. Conclusions

In summary, we demonstrated a facile and novel CMCNFs-PEI adsorbent that has a high adsorption capacity for Cu^2+^. Compared with pure CMCNF, the hydrophilicity of CMCNF-PEI obviously decreased, and this led to an appearance of a 3D network structure. Remarkably, adsorption isotherm data revealed that the maximum adsorption capacity for Cu^2+^ was in the order: CMCNC30-PEI (618.48 mg/g) > CMCNF20-PEI (528.36 mg/g) > CMCNF10-PEI (307.43 mg/g). Kinetic studies showed that the adsorption process of Cu^2+^ on CMCNFs-PEI was more likely to be modeled by pseudo-second-order equation. The adsorption mechanism of Cu^2+^ might be attributed to the active sites (carboxyl, amino groups) on the surface of CMCNF30-PEI. In addition, EDTA solution can regenerate CMCNF30-PEI, with the adsorption capacity still retained at 85% after three cycles. This work provides a facile and novel method for preparing the CMCNFs-based Cu^2+^ adsorbent.

## Figures and Tables

**Table 1:** Thermal stability parameters of EBP and CMCNFs.

	EBP	CMCNF10	CMCNF20	CMCNF30	CMCNF10-PEI	CMCNF20-PEI	CMCNF30-PEI
Carboxyl content (mmol/g)		0.99	1.52	2.01			
T_0_ (°C)	276.5	267.99	225.71	232.96	218.64	219.60	218.19
T_max_ (°C)	388.78	353.76	340.49	330.78	380.09	380.36	381.50

T_0_, T_max_ were calculated from TGA curves.

**Table 2:** Specific surface area and average pore size of CMCNFs-PEI aerogel.

Sample	Bet Surface Area (m^2^/g)	Langmuir Surface Area (m^2^/g)	Pore Size (Å)
CMCNF10-PEI	0.82	0.21	92.85
CMCNF20-PEI	1.16	1.55	121.8
CMCNF30-PEI	1.52	2.15	163.3

**Table 3:** Kinetic and isotherm parameters for Cu^2+^ adsorption onto CMCNFs-PEI aerogels.

Model	Parameters	Aerogels		
		CMCNF10-PEI	CMCNF20-PEI	CMCNF30-PEI
1st-order kinetic	*k*_1_ (g/mg∙h)	0.063	0.096	0.1086
	*q_exp_* (mg/g)	15.26	16.62	18.67
	*q_cal_* (mg/g)	14.50	15.31	16.99
	*R* ^2^	0.9470	0.9060	0.9387
2nd-order kinetic	*k*_2_ (g/mg∙h)	0.0059	0.0074	0.0078
	*q_exp_* (mg/g)	15.26	16.62	18.67
	*q_cal_* (mg/g)	15.51	16.48	18.18
	*R* ^2^	0.9874	0.9652	0.9745
Langmuir isotherm	*K_L_* (L/g)	0.0036	0.0019	0.0013
	*q*_0_ (mg/g)	307.43	528.36	616.48
	*R* ^2^	0.9845	0.9915	0.9972
Freundlich isotherm	*K_F_*	82.07	62.18	114.02
	*n*	4.13	2.74	5.74
	*R* ^2^	0.9843	0.9933	0.9845

**Table 4:** Thermodynamic parameters of CMCNF30-PEI adsorption for Cu^2+^.

T(K)	∆G (KJ/mol)	∆H (KJ/mol)	∆S (KJ/mol/K)
288	−20.6	49.33	0.2428
298	−23.02		
308	−25.45		

**Table 5:** Comparison of Cu^2+^ adsorption capacity of different materials.

Adsorbent	Adsorption Capacity (mg/g)	Cycle Times	Ref
DNFCs	29.52		[22]
TO-CNF/PVA/PEI nanoparticles	156.8	3	[16]
Fe_3_O_4_@zeolite NaA	86.54		[31]
CPCNCs	131.16	4	[32]
CMCNF30-PEI	614.48	3	This work
